# Genome-Wide Analyses of *SlFWL* Family Genes and Their Expression Profiles under Cold, Heat, Salt and Drought Stress in Tomato

**DOI:** 10.3390/ijms241411783

**Published:** 2023-07-22

**Authors:** Chunxia Ran, Yingying Zhang, Feifei Chang, Xuedong Yang, Yahui Liu, Quanhua Wang, Weimin Zhu

**Affiliations:** 1 Shanghai Collaborative Innovation Center of Plant Germplasm Resources Development, College of Life Sciences, Shanghai Normal University, Shanghai 200234, China; chunxiaran2020@163.com (C.R.); zhangyingying2018@saas.sh.cn (Y.Z.); 2 Shanghai Key Laboratory of Protected Horticulture Technology, The Protected Horticulture Institute, Shanghai Academy of Agricultural Sciences, Shanghai 201403, China; changfeifei12@163.com (F.C.); yxuedong@hotmail.com (X.Y.); liuyahui@saas.sh.cn (Y.L.)

**Keywords:** tomato (*Solanum lycopersicum*), *FWL* (*FW2.2-like*) gene family, PLAC8 structural domain, phylogenetic analysis, expression analysis

## Abstract

PLAC8 is a cysteine-rich protein that serves as a central mediator of tumor evolution in mammals. PLAC8 motif-containing proteins widely distribute in fungi, algae, higher plants and animals that have been described to be implicated in fruit size, cell number and the transport of heavy metals such as cadmium or zinc. In tomatoes, *FW2.2* is a PLAC8 motif-containing gene that negatively controls fruit size by regulating cell division and expansion in the carpel ovary during fruit development. However, despite *FW2.2*, other *FWL (FW2.2-Like)* genes in tomatoes have not been investigated. In this study, we identified the 21 *SlFWL* genes, including *FW2.2*, examined their expression profiles under various abiotic adversity-related conditions. The *SlFWL* gene structures and motif compositions are conserved, indicating that tomato *SlFWL* genes may have similar roles. Cis-acting element analysis revealed that the *SlFWL* genes may participate in light and abiotic stress responses, and they also interacted with a variety of phytohormone-responsive proteins and plant development elements. Phylogenetic analyses were performed on five additional plant species, including *Arabidopsis*, pepper, soybean, rice and maize, these genes were classified into five subfamilies. Based on the results of collinearity analyses, the *SlFWL* genes have a tighter homologous evolutionary relationship with soybean, and these orthologous *FWL* gene pairs might have the common ancestor. Expression profiling of *SlFWL* genes show that they were all responsive to abiotic stresses, each subgroup of genes exhibited a different expression trend. Our findings provide a strong foundation for investigating the function and abiotic stress responses of the *SlFWL* family genes.

## 1. Introduction

*FW2.2* (*fruit weight 2.2*), the first locus identified as a significant QTL regulating tomato fruit weight, controls tomato fruit size by negatively regulating the number of cell divisions in the carpel ovary [1,2]. It plays a crucial role in the domestication and agronomic advancement of tomatoes and contributes up to 30% of the diversity in fruit weight variation, making it a vital factor in the evolution of fruit size [1]. *FW2.2* is a member of a broad family of cysteine-rich eukaryotic proteins with a placenta-specific 8 conserved structure, originally discovered in mammalian placental proteins [3,4]. In tomatoes, the *FW2.2* gene primarily affects fruit size by regulating the number of cell divisions and expansions in the carpel ovary, and the allele variations in the expression of *FW2.2* alleles manifest as differences in transcript levels and timing of expression [5]. Further research has indicated that *FW2.2* also affects other phenotypes, such as fruit number and photosynthate distribution [6]. Furthermore, yeast two-hybrid studies have shown that *FW2.2* physically interacts with the CKII kinase, which is located in or around the plasma membrane and has been extensively studied in animals and yeast cells, where it forms part of the cell cycle-related signal transduction pathway [7].

In addition, *FW2.2* has numerous homologs in plants, animals, and fungi [3,8]. In plants, it has been discovered that *FW2.2-like (FWL)* genes serve significant roles in regulating the size of fruit and cell number [3,9,10]. *CNR1* (*Cell Number Regulator 1*) and *CNR2*, two of the *FW2.2* orthologs in maize, act as negative growth regulators, reducing the size of the entire plant and its organs when *CNR1* is overexpressed, whereas the expression of *CNR2* was shown to be inversely linked with the vigor of hybrid seedlings and tissue growth activity [3]. The known *FWL* gene family in rice has eight members. Among them, *OsFWL3* has a negative correlation with the glume’s growth activity, the grain length of the *osfwl3* mutant was longer than the wild type. Analysis of GUS activity revealed that the expression level of *OsFWL3* in mature glume was significantly higher than that in developing glume. While *OsFWL5* negatively regulated plant height, the plant height of the *osfwl5* mutant was distinctly shorter than that of the control [11]. Numerous plant genes have been revealed to be involved in the development of root nodules, which take place when compatible rhizobia infect the root hairs of plants and induce cortical plant cell division to occur rapidly [12]. A homolog of the *FW2.2* gene was significantly up-regulated during nodule development and was named *GmFWL1* [8,13]. Moreover, the cysteine-rich PLAC8 domain has also been linked to heavy metal resistance in plants, and the membrane protein family member PCR (plant cadmium resistance) protein imparts cadmium tolerance [14,15,16]. Many members of the *FWL* gene family play significant roles in the translocation of cadmium and zinc [3,11,14,17]. *OsFWL4* expression in rice seedlings affects Cd resistance, when exposed to cadmium, the expression of *OsFWL4* in rice seedlings considerably changed in their root and stem tissues, whereas their Cd concentration was significantly reduced in their above-ground tissues [18]. *OsFWL5/OsPCR1* and *OsFWL2/OsPCR3* are closely associated with Cd accumulation. The cadmium buildup in the rice roots, stems, leaves and seeds was greatly enhanced in the RNAi plants of *OsPCR1* and *OsPCR3*, however, this was not the case in the overexpressed plants, which displayed cadmium tolerance [19]. In *Arabidopsis thaliana*, the Cys-rich membrane protein AtPCR1 was first obtained from the cadmium-sensitive yeast strain ycf1, and overexpressing *AtPCR1* exhibited increased Cd(II) resistance [15]. A yeast growth assay showed that yeast strains expressing *AtPcr1*, *2*, *9* and *10* grew better than empty vector-expressing strains on Cd(II)-containing plates [15]. AtPCR2 has been identified as a crucial zinc transporter responsible for maintaining optimal zinc levels in *Arabidopsis*. Mutants with a loss of *atpcr2* function are sensitive to zinc stress under zinc excess and deficiency conditions [20]. Similarly, *OsFWL5/OsPCR1* expression was found to increase during seed development in *GW2* (*Grain Weight 2*) loss-of-function mutants, which encode a protein that regulates seed weight [21]. *OsFWL5/OsPCR1* was found to be involved in both the control of controlling grain weight, length and breadth as well as for the resistance of rice to heavy metals like zinc, cadmium and lead, according to analysis of the *OsFWL5/OsPCR1* knockout and overexpression lines [21,22]. It was discovered that *MCA1* and *MCA2*, two members of the Arabidopsis *FWL* family with PLAC8 motifs, are situated at the plasma membrane and take up Ca^2+^ as well as mediate the rise in cytosolic Ca^2+^, which is generated by cold and the development of cold tolerance in Arabidopsis [23,24]. These FWL proteins share many structural features, including a potential transmembrane (TM) fragment, an EF hand-like region in the N-terminal half, a coiled helical motif in the middle, and a PLAC8 motif [24,25].

Tomato (*Solanum lycopersicum*), a widely cultivated vegetable crop, is highly susceptible to abiotic stresses, which can affect plant growth, reduce photosynthetic rate, disrupt ion balance and yield [26]. However, the *FWL* family members in tomatoes have not been well characterized, and few studies have been reported on their response to various abiotic stresses. In this study, we characterized the *SlFWL* gene family in tomatoes and performed a comprehensive analysis of their protein physicochemical properties, chromosomal localization, gene structure, protein motifs, phylogenetic relationships and subcellular localization predictions. We also analyzed their expression profiles in response to abiotic stress such as low temperature (4 °C), high temperature (42 °C), salt (NaCl) and drought (PEG) stimuli. This study provides a better understanding of the structural characteristics of tomato *SlFWL* genes and serves as a basis for the functional validation of *SlFWL* genes and their role in stress tolerance in tomato plants.

## 2. Results

### 2.1. Identification of the SlFWL Genes in Tomato

To identify the members of the *FWL* gene family in tomatoes, we conducted HMM searches using the amino acid sequences in tomato, followed by BLASTP to detect any missing *SlFWL* genes. We further validated the conserved structural domain, PF04749, using NCBI-CDD. In total, we identified 21 *SlFWL* genes in tomatoes, including *FW2.2*. These genes were named based on their respective chromosome numbering. The basic characteristics of these genes and their corresponding proteins were analyzed (Table 1). The majority of *SlFWL* genes encode short peptides with 100–300 amino acids, while only a few contain more than 400 amino acids. Their molecular weights range from 11.07 to 57.09 kDa, their isoelectric points range from 4.48 to 9.37, and their cysteine content spans from 2.7% to 12.8%. Most of the *SlFWL* genes were predicted to localize in the cell membrane, except for SlFWL6, which was predicted to localize in the nucleus. *SlFWL2*, *SlFWL3*, *SlFWL10*, *SlFWL11* and *SlFWL14* were predicted to localize in both the cell membrane and nucleus. The transmembrane structure prediction results show that more than half of the SlFWL proteins have at least one transmembrane structure. The various predictions of subcellular localization and the number of transmembrane structures may indicate the functional diversity of SlFWL proteins.

### 2.2. Chromosomal Localization, Phylogenetic Relationships, and Gene Structures of the SlFWL Genes

**Figure 1:**
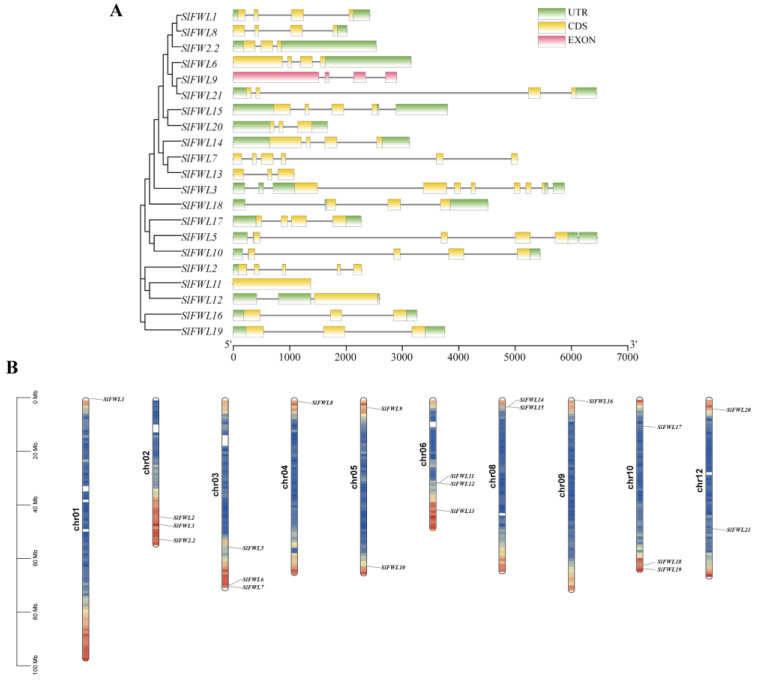
Phylogenetic tree, gene structure, and chromosome distribution of *SlFWLs.* (**A**) Phylogenetic tree and gene structure analysis of the *SlFWLs.* Using the neighbor-joining (NJ) method with the bootstrap method’s default parameters set to 1000, the Poisson model on, and MEGA (Version 11). Numbers below the branches denote frequencies, whereas numbers surrounding the nodes denote branch lengths. UTR sequences and CDS are shown by green and yellow boxes, respectively. Non-protein-encoding exons are represented by pink boxes, and introns are represented by lines. (**B**) Chromosome distribution of *SlFWL* genes.

To better understand the evolutionary relationships among the *SlFWL* genes, we generated a phylogenetic tree using the neighbor-joining (NJ) method and visualized the gene structure using TBtools (v1.09876) (Figure 1A). The chromosomal localization analysis revealed that all *SlFWL* genes were tandemly and relatively evenly distributed on 10 chromosomes of tomatoes, with at least one *SlFWL* gene on each chromosome (Figure 1B). The sequence similarity of the tandemly distributed *SlFWL* genes was not very high, as demonstrated by the phylogenetic tree and chromosomal localization, indicating that they are unlikely to have common functions despite being physically adjacent. The intron and exon architecture of the *SlFWL* genes show that their exons were relatively short, and their distribution traits were comparable to those of *FW2.2*. *SlFWL9* is a pseudogene, as it lacks a coding region. Each *SlFWL* gene has one to seven exons, and the distribution of the coding region of most *SlFWL* genes was conserved. SlFWL11 and *SlFWL12* only contain 1 exon, *SlFWL3* and *SlFWL7* have six to seven exons, and other members have a fairly consistent distribution of 3–4 exons. The diversity of the gene structure of the *SlFWL* family genes suggests a variety of putative biological activities.

### 2.3. Conserved Motif Analysis of SlFWL Genes

**Figure 2:**
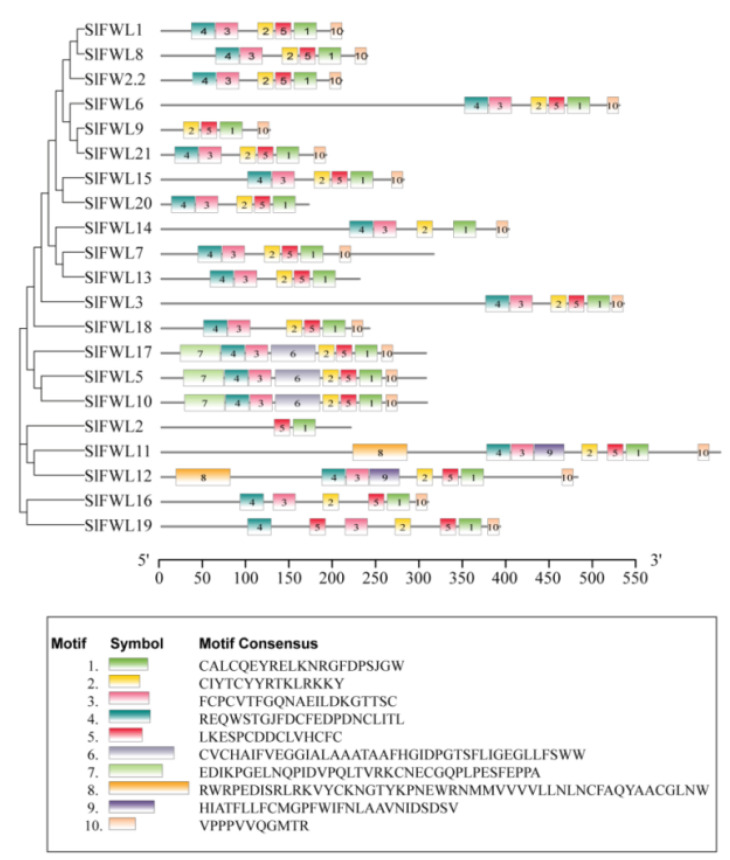
Conserved motif analysis of SlFWL proteins. Different conserved motifs are indicated by numbers and different colors. Protein sequences of these motifs are displayed below.

The *SlFWL* genes in tomatoes were analyzed for conserved motifs using MEME (https://meme-suite.org/meme/tools/meme, accessed on 16 May 2022), with the identification of 10 conserved motifs (Figure 2). With the exception of SlFWL2 and SlFWL9, most SlFWL proteins were found to have similar conserved motifs, including motifs 1, 2, 3, 4 and 5. Motifs 3, 4 and 5 were identified as conserved sequences of the PLAC8 protein. Interestingly, motifs 3 and 4 were located at the N-terminus of these SlFWL proteins, while motif 5 was located at the C-terminus. Most SlFWL proteins also had motif 10, with only SlFWL2 and SlFWL13 lacking it. These results suggest that SlFWL proteins containing the PLAC8 structural domain were conserved during evolution. Additionally, SlFWL5, SlFWL10 and SlFWL17 had motifs 6 and 7, in which motif 7 contained a conserved sequence of a zinc finger domain. Motifs 8 and 9 were only present in SlFWL11 and SlFWL12. These results imply that the SlFWL proteins might have a relatively conserved function and have diverged during evolution.

### 2.4. Analysis of Cis-Regulatory Elements in SlFWL Genes

**Figure 3:**
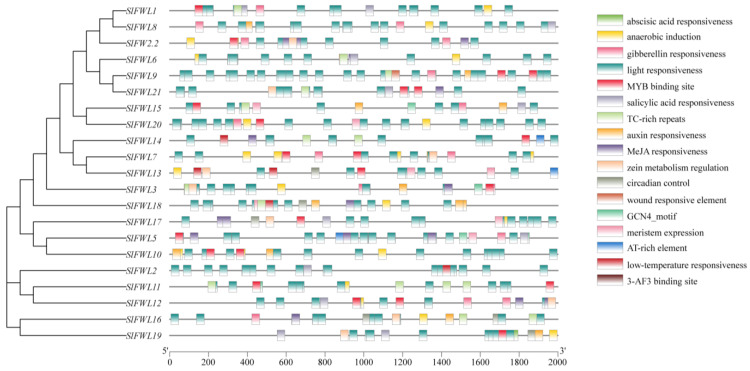
Analysis of cis-regulatory elements of the *SlFWL* genes. Different elements are indicated by different colors.

The 2 kb upstream sequences from the translation start sites of the *SlFWL* genes were analyzed to identify their cis-regulatory elements and investigate their potential regulatory mechanisms in plant growth, development, and abiotic stress responses (Figure 3, Table S1). The analysis revealed that the promoter regions of the *SlFWL* genes contained a large number of light response elements, with a total of 278 elements identified. Moreover, most of the *SlFWL* genes contained phytohormone elements, such as abscisic acid response elements, jasmonic acid response elements, and salicylic acid response elements, as well as gibberellin response elements. Additionally, a total of 22 MYB binding sites were detected, which also contained seven rhythm-related elements and six low-temperature response elements. In addition, anaerobic response elements, defense stress response elements, TC-rich repeats (defense and stress), GCN4_motif (sperm endosperm), and zein metabolism regulation response elements were also detected. These findings suggested that the *SlFWL* genes may participate in many complex regulatory pathways involved in plant development, metabolic processes, and stress responses.

### 2.5. Homology Analysis of FWL Genes from Different Species

**Figure 4:**
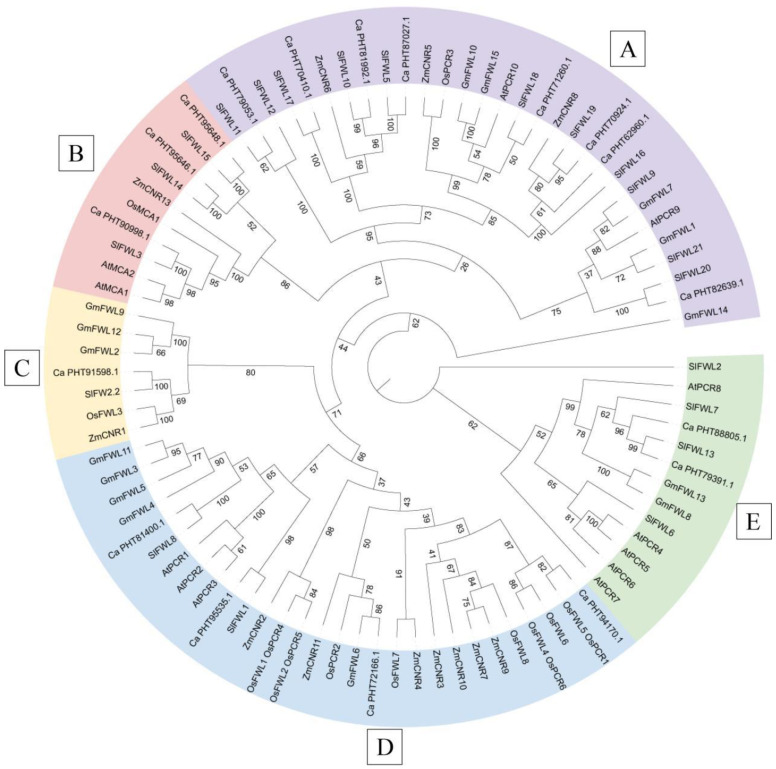
Homology analysis of FWL proteins in different species. Multiple sequence alignments of FWL proteins were performed using the MUSCLE alignment function in MEGA (Version 11) with default settings. The maximum-likelihood method of the IQ-TREE function in TBtools (v1.09876) software was applied to construct the phylogenetic tree with 1000 bootstrap replicates. The FWL proteins were used to run 1000 self-replicates and build a phylogenetic tree using the neighbor-joining method on the MEGA (Version 11) program. Based on branching, the *FWL* family proteins were classified into five subfamilies. Each subfamily is represented by a distinct color: purple for subfamily A, red for subfamily B, yellow for subfamily C, blue for subfamily D, and green for subfamily E. Sl (*Solanum lycopersicum*) stands for tomato, At (*Arabidopsis thaliana*) for Arabidopsis, Ca (*Capsicum annuum*) for pepper, Gm (*Glycine max*) for soybean, Os (*Oryza sativa*) for rice and Zm (*Zea mays*) for maize.

In order to investigate the homology of FWL proteins across different plant species, FWL proteins from 68 other species were extracted from EnsemblPlant, including proteins from dicotyledonous plants such as *Arabidopsis*, pepper and soybean, as well as monocotyledonous plants such as rice and maize [3,10,13,15,27]. A multispecies phylogenetic tree was generated (Figure 4, Table S2). Based on the branching of the evolutionary tree, the FWL protein family was divided into five subfamilies. All five subfamilies are present in both monocots and dicots. Subfamily E contained most AtPCR members, with SlFWL2 located on a specific subbranch in this subfamily. SlFWL5, SlFWL9, SlFWL10, SlFWL11, SlFWL12, SlFWL16, SlFWL17, SlFWL18, SlFWL19, SlFWL20 and SlFWL21 were closely related to OsPCR3, ZmCNR5, ZmCNR6, ZmCNR8, GmFWL1, AtPCR9 and AtPCR10, belonged to subfamily A. The members of subfamily B were mainly MCA, SlFWL14 and SlFWL15 were also located in this family. Tomato FW2.2 was the only FWL member in subfamily C, which also included pepper PHT91598.1 (Cell number regulator 1) protein. SlFWL1 and SlFWL8 exist in subfamily D, which also contains AtPCR1, AtPCR2, AtPCR3, OsPCR1, OsPCR4, OsPCR5 and OsPCR6. These results suggest that FWL proteins are widely existent and diverse across plant species and exhibit sequence conservation in related species during evolution. This implies that FWL proteins may perform similar biological functions among different species.

### 2.6. Collinearity Analysis of SlFWL Genes with Other Species

**Figure 5:**
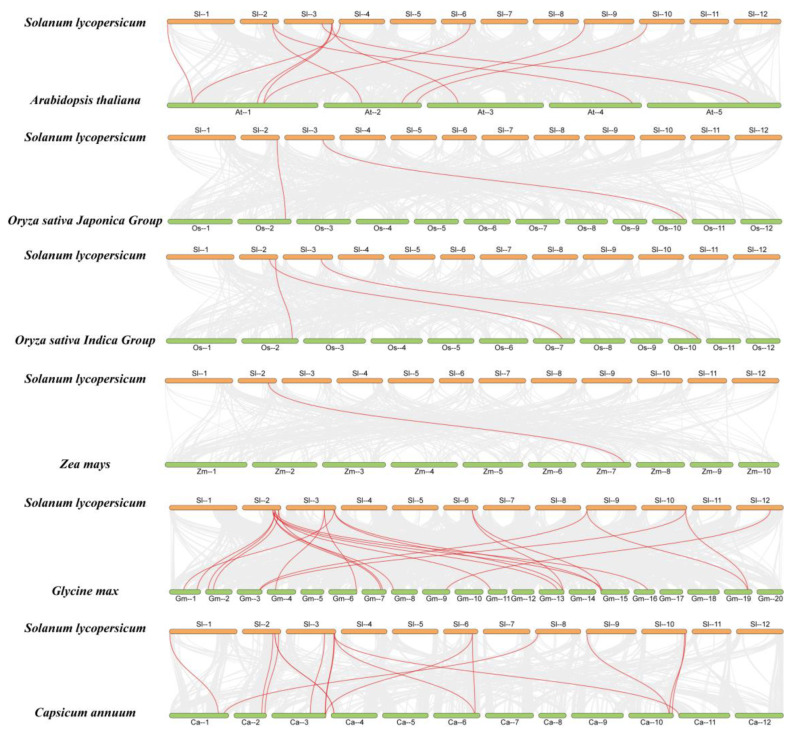
Collinearity analysis of *SlFWL* genes with other species. The gray line in the background represents the collinearity between tomatoes and the other five plant genomes (*Arabidopsis thaliana*, *Oryza sativa*, *Zea mays*, *Glycine max* and *Capsicum annuum*), while the red line emphasizes the synteny gene pairs.

The extension and evolution of gene families in the genome are significantly influenced by tandem and fragmental replication between gene families, and tandem replication is a key mechanism for creating new copies of genes [28]. To investigate the evolutionary relationships of *FWL* genes in different plant species, we performed a synteny analysis of *FWL* genes between the genomes of tomato, *Arabidopsis*, rice (*Oryza indica* and *Oryza japonica*), maize, soybean and pepper (Figure 5, Table S3). The results show that the number of collinear gene pairs between tomato and the other species ranged from 1 to 22, suggesting that these collinear genes might originate from the same ancestor and are functionally conserved. The highest number of collinear genes was found between the tomato and soybean genomes, while maize and rice had the lowest number of collinear genes. These findings indicate that *FWL* genes might have undergone a complex evolutionary history.

### 2.7. Expression Profile Analysis of SlFWL Genes

**Figure 6:**
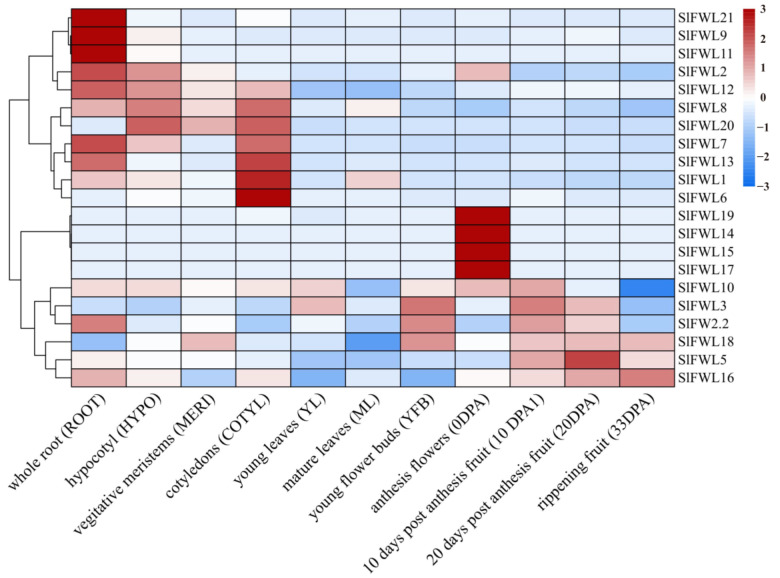
Expression pattern of *SlFWL* genes in different tissues.

Expression profiles of *SlFWL* genes were analyzed using data from the public RNA-seq database (http://ted.bti.cornell.edu/cgi-bin/TFGD/digital/home.cgi, accessed on 18 May 2022). The results show that each *SlFWL* gene exhibited a distinct tissue expression pattern (Figure 6, Table S4). *SlFWL2*, *SlFWL9*, *SlFWL11*, *SlFWL12* and *SlFWL21* were abundantly expressed in roots, followed by hypocotyl, and had a comparatively low expression level throughout the fruiting stage. Except for *SlFWL20*, all *SlFWL* genes were expressed in roots. *SlFWL1*, *SlFWL6*, *SlFWL7*, *SlFWL8*, *SlFWL13* and *SlFWL20* were mainly expressed in cotyledons. *SlFWL14*, *SlFWL15*, *SlFWL17* and *SlFWL19* were specifically expressed in mature flowers. *FW2.2*, *SlFWL3*, *SlFWL5*, *SlFWL10*, *SlFWL16* and *SlFWL18* show different expression patterns in various tissues. The tissue-specific expression patterns of *SlFWL* genes suggest that they may be involved in different biological processes and have diverse functions. The abundant expression of *SlFWL* genes in roots indicates their potential role in root development and response to environmental stresses, which is consistent with the presence of stress-related elements in their promoter regions. The expression of *SlFWL* genes in different tissues also suggests their possible roles in plant growth and development, as well as in response to various internal and external cues. These results provide a foundation for further investigation of the functions of *SlFWL* genes in tomatoes and other plant species.

### 2.8. Expression of SlFWL Genes in Response to Abiotic Stress

**Figure 7:**
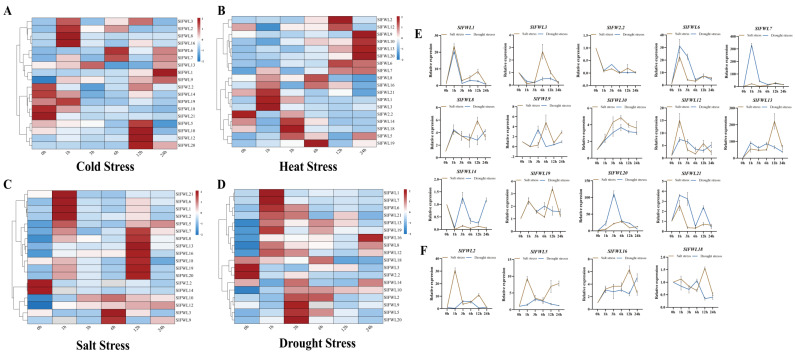
qPCR analysis of *SlFWL* gene expression under abiotic stress treatments. (**A**) Cold stress; (**B**) Heat stress; (**C**) Salt stress (NaCl); (**D**) Drought stress (PEG6000); (**E**,**F**) The expression trends of *SlFWL* genes between drought stress and salt stress. The standard deviations are shown with error bars. Different letters indicate significant differences (*p* < 0.05).

To investigate the function of the *SlFWL* gene family under abiotic stress, we examined the expression patterns of *SlFWL* genes in tomato leaves subjected to cold, heat, salt, and drought stresses at 0, 1, 3, 6, 12 and 24 h after stress treatments. *FW2.2* and other *SlFWL* genes exhibited diverse expression patterns over time following various stress treatments, while *SlFWL11*, *SlFWL15* and *SlFWL17* were undetectable in plants under either control or abiotic stress treatments (Figure 7 and Figures S1–S4, Table S6).

Under cold stress, the majority of *SlFWL* genes were up-regulated. *SlFWL2*, *SlFWL3*, *SlFWL6*, *SlFWL7*, *SlFWL8*, *SlFWL9*, *SlFWL12*, *SlFWL13*, *SlFWL16* and *SlFWL19* exhibited a similar expression trend in response to cold stress, with a significant increase at 1 h of cold stress treatment, followed by a downward trend at 3 h or 6 h (Figure 7A and Figure S1, Table S6). Notably, *SlFWL2* increased by 28-fold in response to cold stress. However, the expression of *SlFWL1* was remarkably different, showing a 150-fold increase in expression after 24 h of exposure to cold stress. Short-term cold stress treatment dramatically reduced the expression of *FW2.2*, *SlFWL5*, *SlFWL10* and *SlFWL14*, after 12 h, their expression began to climb. Under cold stress, the expression of *SlFWL18* and *SlFWL21* constantly decreased. *SlFWL20* did not respond to cold stress until 6 h, and its expression considerably increased by 10 times at 12 h.

Heat stress significantly altered the transcriptional profile of *SlFWL* genes (Figure 7B and Figure S2, Table S6). *SlFWL1*, *SlFWL2*, *SlFWL3*, *SlFWL5*, *SlFWL7*, *SlFWL8*, *SlFWL13*, *SlFWL16* and *SlFWL21* exhibited a considerable increase in expression (1.25–59-fold) after 1 h of heat treatment in comparison to the control (0 h). Under heat stress, the expression of *SlFWL1* instantly rose 59-fold at 1 h. *SlFWL19* did not respond to heat stress before 6 h, and then reached its highest expression level. The response of *SlFWL20* to heat stress lasted long and was consistently up-regulated within 24 h. The expression of *SlFWL6*, *SlFWL9*, *SlFWL10* and *SlFWL12* was down-regulated in the short term but peaked within 6–12 h under heat stress. *FW2.2*, *SlFWL14* and *SlFWL18* were down-regulated at 1 h under heat stress, and, after a brief recovery, their expression was significantly suppressed after 6 h.

In addition, most *SlFWL* genes responded to salt stress treatment (Figure 7C and Figure S3, Table S6). Among these genes, *SlFWL1*, *SlFWL2*, *SlFWL5*, *SlFWL6*, *SlFWL7*, *SlFWL8*, *SlFWL10*, *SlFWL12*, *SlFWL13*, *SlFWL16*, *SlFWL18*, *SlFWL19*, *SlFWL20* and *SlFWL21* all exhibited varying degrees of increased expression. Within 1–12 h of salt stress treatment, *SlFWL1*, *SlFWL2*, *SlFWL6*, *SlFWL7*, *SlFWL13* and *SlFWL20* show substantial increases, with the expression level increasing more than 20-fold. Notably, *SlFWL13* shows the most significant increase, with expression levels increasing more than 200-fold after 12 h of treatment. The expression of *SlFWL3* and *SlFWL9* reached its peak at 6 h of salt stress. However, *FW2.2* and *SlFWL14* were barely detectable after 24 h of salt stress treatment, indicating that salt stress inhibited their expression.

Under drought and salt stress, *SlFWL* genes had similar expression profiles. For example, *FW2.2*, *SlFWL1*, *SlFWL3*, *SlFWL6*, *SlFWL7*, *SlFWL8*, *SlFWL9*, *SlFWL10*, *SlFWL12*, *SlFWL13*, *SlFWL14*, *SlFWL19*, *SlFWL20* and *SlFWL21* shared a common expression pattern under both stresses, almost with a simultaneous increase or decrease in expression (Figure 7C–F, Figures S3 and S4, Table S6). Intriguingly, among them, some genes, such as *FW2.2*, *SlFWL2*, *SlFWL3*, *SlFWL9*, *SlFWL14* and *SlFWL18*, were repressed by drought stress in the early stage, while others were significantly induced by drought stress and responded strongly at different time points (Figure 7E,F).

In summary, the study provides evidence for the differential expression of *SlFWL* genes under abiotic stress treatments. These findings could be helpful in developing strategies to improve the tolerance of crops to these environmental stresses.

## 3. Discussion

FWL proteins with the PLAC8 structural domain are known to be universally present in plants, mammals, and fungi and have been found to play critical roles in regulating plant organ size, metal ion homeostasis and root tumor formation [1,3,8,11]. Several *FWL* genes in different species have been identified, including *GmFWL1*, *OsFWL3*, *ZmCNR3*, *MdCNR8*, *cell number regulators* (*CNRs*) and *Pafw2.2-like* [3,8,11,17,29,30]. These *FWL* genes play a conserved role in repressing cell division or controlling cell number to control plant organ size [17]. However, it is yet unknown whether tomato *FWLs* react to abiotic stress. In this study, we performed a genome-wide identification of the tomato *FWL* (*FW2.2-Like*) genes and thoroughly analyzed the gene structure, chromosomal localization, phylogeny, gene duplication, cis-regulatory elements, expression profiles in different tissues and in response to different abiotic stress treatments in tomatoes.

In this study, we systematically identified a total of 21 *SlFWL* genes, including the well-known gene *FW2.2*, which is distributed on 10 chromosomes in tomatoes (Figure 1). Among them, *SlFWL6* and *SlFWL7*, *SlFWL11* and *SlFWL12*, *SlFWL13* and *SlFWL14*, and *SlFWL18* and *SlFWL19* are connected two by two tandemly on ch03, ch06, ch08 and ch10, respectively. *SlFWL* genes exhibit a degree of conservation and segregation in physicochemical properties, such as amino acid length, molecular weight and isoelectric point. These SlFWL proteins range in size from 98 to 505 amino acids, but most are relatively short, between 100 and 300 amino acids. Each SlFWL protein contains an average of 6.7% Cys, and the conserved portion rich in cysteine is sometimes referred to as the PLAC8 motif (Table 1) [3].

Our anticipated subcellular localizations demonstrate that the *SlFWLs*, as well as *FW2.2* and other *FWL* genes in other plants, are primarily distributed on the intracellular cell membrane. Compelling evidence suggests that *FW2.2* interacts physically with the regulatory (beta) subunit of a CKII kinase at or near the plasma membrane. This discovery implied that *FW2.2* may serve as a component of a signaling mechanism activated by extracellular signals that regulate fruit cell division [1,7]. Additionally, numerous studies have emphasized plant hormones and their interrelated roles in controlling fruit development and fruit size [31]. The tomato ovary consists of two or more carpels enclosing the locular containing the ovule. After successful fertilization, a period of cell division and cell expansion begins, which continues for 6–7 weeks [32]. During the period of cell division, the auxin concentration is likely to increase, some of which shows peak expression at the stage of cell expansion, suggesting that phytormone auxin plays a role in fruit initiation and fruit-size cell division by regulating cell division and cell expansion processes [33]. Cell proliferation and expansion are known to be regulated by intricate interactions between stimulatory signals, including hormone signaling and carbon partitioning through the activation of D-type cyclins [34]. For instance, *FW2.2* may function in a signaling pathway that connects hormone or sugar signals to the control of cell cycle machinery in developing flowers. This pathway may affect inflorescence number by the modulation of photosynthate partitioning in the plant and may act upstream of several genes associated with cell proliferation, such as *CYCD3; 1* and *KRP* [35]. However, whether *FW2.2* is involved in the distribution and response of plant hormones is unknown, and the molecular mechanism by which *FW2.2* connects hormone and sugar signals to regulate cell proliferation remains an open question.

The majority of members of the *FWL* family are transmembrane domain proteins, and several investigations have shown that they are frequently found localized in the plasma membrane of cells [14]. In other plants, *AtPCR1* was localized at the plasma membrane [14]. The amino acid sequences of AtMCA1 and AtMCA2 share several common structural features, such as putative transmembrane (TM) segments and an EF hand-like region in the N-terminal half. These structural features are crucial for the activity of Ca^2+^ uptake and can function as intact membrane proteins [24]. A TM fragment with the CC(L)XXXXCPC domain forms a pore with the hydrophilic side toward the lumen, cysteine residues are lined on the side toward the lumen, and metal ions can migrate through or interact with the channel [3]. Our transmembrane structure predictions show that more than half of the SlFWL proteins have at least 1 transmembrane structure with an N-terminal PLAC8 structural domain of CC(I/L/W/F/V)XXXXCPC with a change in the second amino acid C (Table S2). There are some *SlFWL* genes that do not contain this structure. We speculated that the function of these genes may have also changed during evolution, which remains to be further verified. It is worth mentioning that cadmium resistance can be achieved by solely expressing the AtPCR1 N-terminal hydrophobic segment, which carries a CCXXXXCPC sequence conserved among various organisms [14].

Differences in the functional expression of genes are inextricably linked to their structure [36]. The structural distribution characteristics of *SlFWL* genes are very similar, except for *SlFWL9*, which has no coding region. The position and distribution of the coding region CDS of all *SlFWL* genes are relatively conserved (Figure 1A). Most of the members have 3–4 exons and are relatively evenly distributed. In contrast, *SlFWL3* and *SlFWL7* have longer genome sequences and more introns than the other genes, implying that they may be early *FWL* family members that achieve transcriptional diversification through processes such as selective splicing to regulate more complex and broad functions [37]. Further analysis of the conserved motif reveals that all SlFWL proteins have very high similarity, with most containing motif 1–5 and motif 10. Motif 3 and motif 4 are at the N-terminus, while motif 1 and motif 5 are distributed at the C-terminus (Figure 2). Motif 5 and motif 1 contain a CXXC sequence, which was exactly a cysteine-rich structural domain of unknown function, known as the PLAC8 or DUF614 motif [3,4,38]. Such structures flanked by cysteines are generally associated with the formation of redox-related proteins, isomerization, and a reduction of disulfide bonds [38]. This feature was consistent with the cysteine morphology of mammalian PLAC8, and this cysteine-rich PLAC8 structure is highly conserved in the *SlFWL* genes. We hypothesized that the PLAC8 structure of the SlFWL protein is also functionally conserved. Common motifs imply functional redundancy, while specific motifs may lead to functional divergence [39]. SlFWL13 and SlFWL20 do not contain motif 10, while motif 7 and motif 6 are unique to SlFWL5, 10 and 17, motif 8 and motif 9 are unique to SlFWL11 and SlFWL12. Pfam searches of the motifs revealed that motif 7 contains a zinc finger domain and an XPA protein N-terminal motif. It is clear that the distribution of *SlFWL* gene structures and conserved motifs forms different clusters, showing some evolutionary similarities and common functions, while the disappearance or appearance of additional motifs and structures may function in the expansion and diversity of the *SlFWL* gene family during evolution. The SlFWL proteins may have functionally diverged during evolution.

Gene duplication results in longer protein sequences, more functional domains, and more cis-regulatory motifs, and gene duplication increases the number of genes and the complexity of gene function [40]. We found a total of 68 associated FWL proteins in dicotyledonous plants (*Arabidopsis*, pepper and soybean) and monocotyledonous plants (rice and maize). These genes are distributed in five major branches (Figure 4). The results suggest that *FWL* is conserved in both monocots and dicots, implying the existence of a common ancestor. From an evolutionary perspective, gene duplication events increased the number of genes in specific gene families, thus enabling plants to adapt and survive under adverse environmental stresses [28,41]. Collinearity analysis revealed that the *SlFWL* genes are most closely related to the soybean genome and have the highest number (22 pairs) of colinear gene pairs between them, followed by pepper (17 pairs) (Figure 5).

We analyzed 2000 bp of cis-regulatory elements (CREs) upstream of the translation start site of *SlFWL* family genes. The promoter region contains multiple light-responsive elements and hormone-related action elements such as abscisic acid-responsive elements, jasmonic acid-responsive elements, gibberellin-responsive elements, and salicylic acid-responsive elements. In addition, these *SlFWL* genes also have stress-signaling elements such as TC-rich MYB- and MYC-binding sites (Figure 4). These results show that *SlFWL* expression is closely associated with abiotic stress and hormone signaling responses. Earlier studies identified a conserved cis-acting regulatory element in the promoter of drought-inducible genes called the ABA response element (ABRE) [42]. MYB proteins are key factors in the regulatory network controlling development, metabolism, and responses to biotic and abiotic stresses, and several stress-related MYB elements have been reported in stress resistance gene promoters [43,44]. Our findings reveal that the majority of *SlFWL* members feature MYB-binding elements. Additionally, the *SlFWL* gene family encompasses the GCN4 motif, which is developmentally significant and appears in numerous seed storage protein genes. Studies have demonstrated that promoter fragments carrying the GCN4 motif serve to regulate the seed-specific expression of genes related to germination [45]. The cis-acting elements suggested that the *SlFWL* genes may play an important role in light response, phytohormone signaling, and stress response.

The expression pattern of *FWL* genes in different tissues has been described in many species. For example, in *Arabidopsis*, reverse transcription PCR (RT-PCR) analysis shows that *MCA2* was expressed in leaves, flowers, roots, angiosperms and stems, the GUS reporter gene for *MCA1* was expressed in cotyledons, leaves, vascular tissues of the main roots and regions of the rosette center corresponding to the stem apical meristem [27]. However, *SlFWL* does not have a uniform gene expression pattern in tomatoes. We obtained each *SlFWL* gene expression pattern in different tissues from public databases (Figure 6, Table S4). Ten *SlFWL* genes were differentially expressed in all tissues of the whole root (ROOT), hypocotyl (HYPO), nutritional meristem (MERI), cotyledon (COTYL), young leaf (YL), mature leaf (ML), young bud (YFB), flower (0 DPA), 10 days post-anthesis fruit (10 DPA), 20 days post-anthesis fruit (20 DPA) and ripe fruit (33 DPA). *SlFWL1*, *2*, *3*, *5*, *10*, *12*, *16* and *19* were highly expressed in all tissues, with higher expression than other *SlFWL* genes. Expression clustering analysis revealed that *SlFWL2*, *9*, *11*, *12* and *21* were highly expressed in roots, followed by hypocotyl, and were relatively low in the fruiting stage. *SlFWL1*, *6*, *7*, *8*, *13* and *20* were more highly expressed in cotyledons and roots than in any other tissues, and *SlFWL14*, *15*, *17* and *19* were specifically expressed in mature flowers. The differential expression of these *SlFWL* genes in different tissues may indicate that they perform different functions in different parts of the plant and remains to be further investigated.

Unfavorable abiotic stress environmental conditions, such as cold, heat, drought and excess salt in the soil, or toxic metals such as aluminum, arsenic, cadmium, etc., are often detrimental or stressful to growth and development, while drought, salt and temperature stress are the main environmental factors that limit the productivity of agricultural plants and threaten food security [46]. Analysis of gene expression patterns during growth and development and exposure to stress stimuli may help determine their functions. Currently, the functions of members of the *FWL* family are focused on fruit and whole plant size and heavy metal resistance and allocation, but roles in abiotic stresses have not been reported [22,47]. In our study, we examined the transcriptional profiles of the *SlFWL* family under cold temperature, high temperature, salt, and drought treatments (Figure 7 and Figure S1–S4, Table S6). *SlFWL11*, *15* and *17* were not detectable in their expression in leaves under either control or abiotic stress treatments, which was consistent with the data from RNA-seq (Table S4). Gene structure analysis revealed that *SlFWL9* is a pseudogene that is able to respond to different stress treatments despite the absence of exons in its gene structure. *FW2.2* was repressed by all stresses, and its expression was significantly reduced under all abiotic stresses. *SlFWL1*, *2*, *3*, *7*, *8*, *13*, *16* and *19* were significantly up-regulated under both cold and heat stress treatments. *SlFWL10*, *14* and *18* were significantly down-regulated under cold and high temperature stresses. *SlFWL5*, *6*, *12*, *20* and *21* show opposite trends in cold and high temperature stress expression trends. *SlFWL1*, *3*, *6*, *7*, *8*, *9*, *10*, *12*, *13*, *14*, *19*, *20* and *21* show similar expression profiles under drought conditions with those under salt stress, with a simultaneous increase or decrease in the short term. These studies suggested that members of the *SlFWL* gene family may have different functions in response to different environmental stimuli, and further studies on their role in response to abiotic stress are needed in the future.

The results of the expression profiles of the *SlFWL* genes as well as in response to salt, drought, cold and heat stress treatments reveal that although some *SlFWL* genes are constitutively expressed in various tissues, they are stress-inducible, suggesting that they play multiple roles in different tissues and in response to different abiotic stress conditions. For instance, *SlFWL8* and *SlFWL9* were upregulated in response to salt stress, while *SlFWL5* and *SlFWL15* were upregulated in response to drought stress. Some *SlFWL* genes show tissue-specific expression patterns, such as *SlFWL1*, which was highly expressed in the root and flower, and *SlFWL2*, which was highly expressed in the fruit. These findings suggest that the *SlFWL* genes may have tissue-specific and stress-responsive functions in tomatoes.

## 4. Materials and Methods

### 4.1. Plant Materials and Growth Conditions

The study was performed on tomato cultivar NR21-16, kept in our laboratory. Tomato seedlings were grown in a growth chamber at 25 °C/16 h and 20 °C/8 h photoperiods (light/dark) with a light intensity of 300 μmol·m^−2^ s^−1^ and 60–70% humidity [48].

To investigate the expression pattern of the *SlFWL* genes under different abiotic stress conditions (cold, heat, salt [NaCl], and drought [PEG]), we subjected 30-day-old (four-leaf-stage) tomato plants with relatively uniform growth conditions to stress treatments for three days and collected leaf samples at 0, 1, 3, 6, 12, and 24 h after the onset of stress [49]. Plants were incubated in growth chambers at 4 °C and 42 °C to apply cold and heat stress, respectively. Salt stress was applied by submerging the roots in a 200 mM NaCl solution [49]. For the drought treatment, PEG6000 was used to simulate drought, and the final concentration of PEG6000 in the hydroponic medium was 12%, and the roots needed to be fully submerged in the PEG6000 solution [50]. The third completely developed leaf of the treatment was collected. Plants with three independent biological seedlings in good condition were used for each sample. Leaf tissues from each biological replicate were collected and mixed thoroughly, then immediately frozen in liquid nitrogen and stored at –80 °C for RNA extraction.

### 4.2. RNA Extraction and Expression Analysis

Total RNA from tomato leaves was extracted using the Biospin Plant Total RNA Extraction Kit (Hangzhou Borui Technology Company, Hangzhou, China) and then reverse transcribed to obtain cDNA by the HiScript II One Step RT-PCR Kit [51]. Quantitative real-time PCR was performed with Hieff UNICON^®^ Universal Blue qPCR SYBR Green Master Mix reagent (Yessen Biotechnology Co., Shanghai, China), and primers were designed by NCBI Primer-BLAST (https://www.ncbi.nlm.nih.gov/tools/primer-blast/, accessed on 8 August 2022) (Table S5). The expression level of the tomato eukaryotic initiation factor gene (eiF, Solyc12g096000) was used as an internal control. The relative expression levels of the genes of interest were calculated using the 2^-ΔΔCT^ method. Each reaction was performed in three replicates.

### 4.3. Identification of Tomato SlFWL Genes

Tomato genome, annotations, and protein sequence files (SL3.0) were downloaded from Ensemble Plants (https://plants.ensembl.org/index.html, accessed on 14 April 2022), and the hidden Markov model (HMM) of PLAC8 (PF04749), the *FWL*-conserved structural domain, was obtained from Pfam (http://pfam.xfam.org/, accessed on 14 April 2022) [52]. Then, the *SlFWL* candidate genes were searched in the tomato whole genome sequence using the Simple HMM search function of TBtools (v1.09876) software and filtered with the *E*-value 1 × 10^−5^ [53]. To obtain the complete *SlFWL* family genes, the FWL protein sequences containing the PLAC8 structural domain in *Arabidopsis thaliana* on NCBI were used as a query to perform a BLASTP search in the tomato protein sequence database with a maximum *E*-value of 1 × 10^−5^ to remove low similarity and duplicate sequences to find all remaining possible *SlFWL* genes [54]. Finally, the presence of the PLAC8 conserved structural domain was verified by NCBI CDD (http://www.ncbi.nlm.nih.gov/Structure/cdd/wrpsb.cgi, accessed on 14 April 2022), and the *SlFWL* family genes were confirmed [55]. All identified genes were localized to tomato chromosomes by TBtools (v1.09876) [53]. The calculation of the number of amino acid residues (aa), molecular weight (kD), percentage of Cys (%), and isoelectric point (pI) of protein peptides of the tomato *SlFWL* family genes was obtained by the online tool ExPASy (http://www.expasy.ch/tools/pi_tool.html, accessed on 20 April 2022) [56]. Cell-PLoc 2.0 (http://www.csbio.sjtu.edu.cn/bioinf/Cell-PLoc-2/, accessed on 20 April 2022) and TMHMM Server v.2.0 (http://www.cbs.dtu.dk/services/TMHMM/, accessed on 14 April 2022) were used for prediction of subcellular localization and the transmembrane structure of the *SlFWL* family members [57,58].

### 4.4. Phylogenetic Relationship, Gene Structure, Protein Motif and Cis-Regulatory Element Analysis of the SlFWL Gene Family

All identified *SlFWL* genes in tomatoes were aligned using ClustalW, and the phylogenetic tree of *SlFWL* genes was generated using the neighbor-joining (NJ) method, the 1000 bootstrap method, and the Poisson model on MEGA (Version 11) [59]. The exon-intron gene structures of the *SlFWL* genes were visualized by TBtools (Gene Structure Visualization (from GTF/GFF3 File)) [53]. The conserved structural domain was analyzed in the Pfam database. Conserved motifs were predicted by the MEME Suite tools (http://meme-suite.org, accessed on 16 May 2022), and the number of motif parameters was manually limited to 10 [60]. The promoter regions (2000 bp upstream of ATG) of the *SlFWL* genes were extracted from the tomato genome sequence using TBtools (v1.09876) software [53]. The promoter sequences were submitted to the PlantCARE online database for analysis (http://bioinformatics.psb.ugent, accessed on 12 May 2022), and these cis-regulatory elements were drawn by TBtools (v1.09876) [53,61].

### 4.5. Evolutionary Relationships and Synteny Analysis of SlFWL Genes in Multiple Species

Multiple sequence alignment analyses of the FWL homologous family members in *Arabidopsis thaliana*, rice, maize, pepper and soybean were performed using the MUSCLE alignment function in the MEGA (Version 11) with default settings [59]. The maximum-likelihood method of the IQ-TREE function in the TBtools (v1.09876) software was applied to construct the phylogenetic tree with 1000 bootstrap replicates [53]. Then, the data were visualized and optimized with the online tool Interactive Tree of Life (iTOL) (https://itol.embl.de/, accessed on 26 May 2022) to generate a phylogenetic tree [62]. The collinearity analysis was performed with One Step MCScanX of the TBtools (v1.09876) software [53].

### 4.6. Tissue Expression Pattern of the SlFWL Gene Family

We obtained tissue expression data of the wild tomato variety LA1589 from the publicly available transcriptome database, Tomato Functional Genomics Database (http://ted.bti.cornell.edu, accessed on 18 May 2022), which included the whole root (ROOT), hypocotyl (HYPO), vegetative meristems (MERI), cotyledons (COTYL), young leaves (YL), mature leaves (ML), young flower buds (YFB), anthesis flowers (0 DPA), 10 days post-anthesis fruit (10 DPA), 20 days post anthesis fruit (20 DPA), and ripening fruit (33 DPA) [63]. We retrieved the FPKM (fragments per kilobase per million reads) values representing the expression levels of *SlFWL* genes. The data for each row were normalized and plotted according to the average FPKM value for each gene. Heatmaps were generated using R software (v.4.2.2), and the package “pheatmap” (v.1.0.12) through Hiplot, a comprehensive web service for biomedical data analysis and visualization [64]. The qRT-PCR data for abiotic stresses were also used for heatmap analysis.

### 4.7. Statistical Analysis

Data was collated using Microsoft Excel 2016 and analyzed for statistics and significance using SPSS software 20.0 (IBM, Armonk, NY, USA), with one-way ANOVA and Duncan’s test (*p* < 0.05) indicating a significant difference. Drawing was conducted using GraphPad Prism 9 (GraphPad Software, San Diego, CA, USA).

## 5. Conclusions

The *FW2.2-like* (*FWL*) gene family in tomatoes, including gene structure, chromosomal location, phylogeny, gene duplication, cis-regulatory elements and expression patterns in response to various abiotic stress treatments, is thoroughly analyzed in this paper. These findings imply that *SlFWL* genes may serve a variety of abiotic stress-related purposes. In addition to facilitating future functional research of this gene family in tomatoes and other plant species, the discovery and characterization of *SlFWL* genes in this work may have repercussions for the future creation of stress-tolerant crops.

## Figures and Tables

**Table 1:** Gene structure and protein properties of *SlFWLs* in tomato.

Gene Name	Gene ID	Gene Locus	Protein Length (aa)	pI	Cys (%)	MW (kDa)	Subcellular Location	Number of Predicted TMHs
*SlFWL1*	Solyc01g005470	SL3.0ch01:324932..322628−	164	5.72	11.0%	17.78	Cell membrane.	1
*SlFWL2*	Solyc02g079390	SL3.0chr02:44525091..44527367−	171	5.37	5.8%	18.75	Cell membrane. Nucleus.	0
*SlFWL3*	Solyc02g083540	SL3.0chr02:47452964..47458840−	418	6.82	4.5%	47.78	Cell membrane. Nucleus.	0
*SlFW2.2*	Solyc02g090730	SL3.0chr02:52889654..52892189+	163	7.45	8.0%	18.06	Cell membrane.	0
*SlFWL5*	Solyc03g093200	SL3.0chr03:55818165..55824621+	239	5.13	6.7%	26.39	Cell membrane. Nucleus.	2
*SlFWL6*	Solyc03g119660	SL3.0chr03:69708462..69711618−	414	5.27	2.7%	46.49	Nucleus.	0
*SlFWL7*	Solyc03g120600	SL3.0chr03:70436458..70441501−	246	5.83	3.3%	27.74	Cell membrane.	0
*SlFWL8*	Solyc04g007900	SL3.0chr04:1572329..1574347+	186	8.42	9.1%	20.89	Cell membrane.	1
*SlFWL9*	Solyc05g009620	SL3.0chr05:3818983..3821883+	98	9.37	7.1%	11.07	Cell membrane.	1
*SlFWL10*	Solyc05g051690	SL3.0chr05:62949477..62954921−	240	4.96	6.7%	26.56	Cell membrane. Nucleus.	2
*SlFWL11*	Solyc06g048790	SL3.0chr06:31806456..31807973+	505	8.81	4.4%	57.09	Cell membrane. Nucleus.	6
*SlFWL12*	Solyc06g048810	SL3.0chr06:31834910..31837505+	376	7.47	4.3%	42.59	Cell membrane.	5
*SlFWL13*	Solyc06g066590	SL3.0chr06:41956218..41957295−	179	5.47	5.0%	20.03	Cell membrane.	0
*SlFWL14*	Solyc08g013910	SL3.0chr08:3387226..3390353+	314	9	2.9%	35.60	Cell membrane. Nucleus.	2
*SlFWL15*	Solyc08g013920	SL3.0chr08:3392240..3396041+	219	6.9	7.3%	24.50	Cell membrane.	1
*SlFWL16*	Solyc09g007490	SL3.0chr09:1044294..1047553+	241	5.39	6.6%	26.33	Cell membrane.	0
*SlFWL17*	Solyc10g018920	SL3.0chr10:10876052..10878322−	239	4.48	7.5%	26.31	Cell membrane.	2
*SlFWL18*	Solyc10g081410	SL3.0chr10:62606037..62610553+	188	4.84	8.0%	20.78	Cell membrane.	1
*SlFWL19*	Solyc10g084260	SL3.0chr10:64000881..64004633−	306	7.11	5.6%	33.64	Cell membrane.	0
*SlFWL20*	Solyc12g013570	SL3.0chr12:4412651..4414323+	133	5.8	10.5%	14.61	Cell membrane.	0
*SlFWL21*	Solyc12g037950	SL3.0chr12:49162527..49168971+	149	6.82	12.8%	16.08	Cell membrane.	0

## Data Availability

Data is contained within the article or supplementary material.

## Supplementary Materials

The supporting information can be downloaded at: https://www.mdpi.com/article/10.3390/ijms241411783/s1.
