# Supplementary figures and images for: Genome-Wide Analyses of SlFWL Family Genes and Their Expression Profiles under Cold, Heat, Salt and Drought Stress in Tomato
Source: Int J Mol Sci. 2023 Jul 22;24(14):11783. doi: 10.3390/ijms241411783 (PMC10380795; doi:10.3390/ijms241411783)

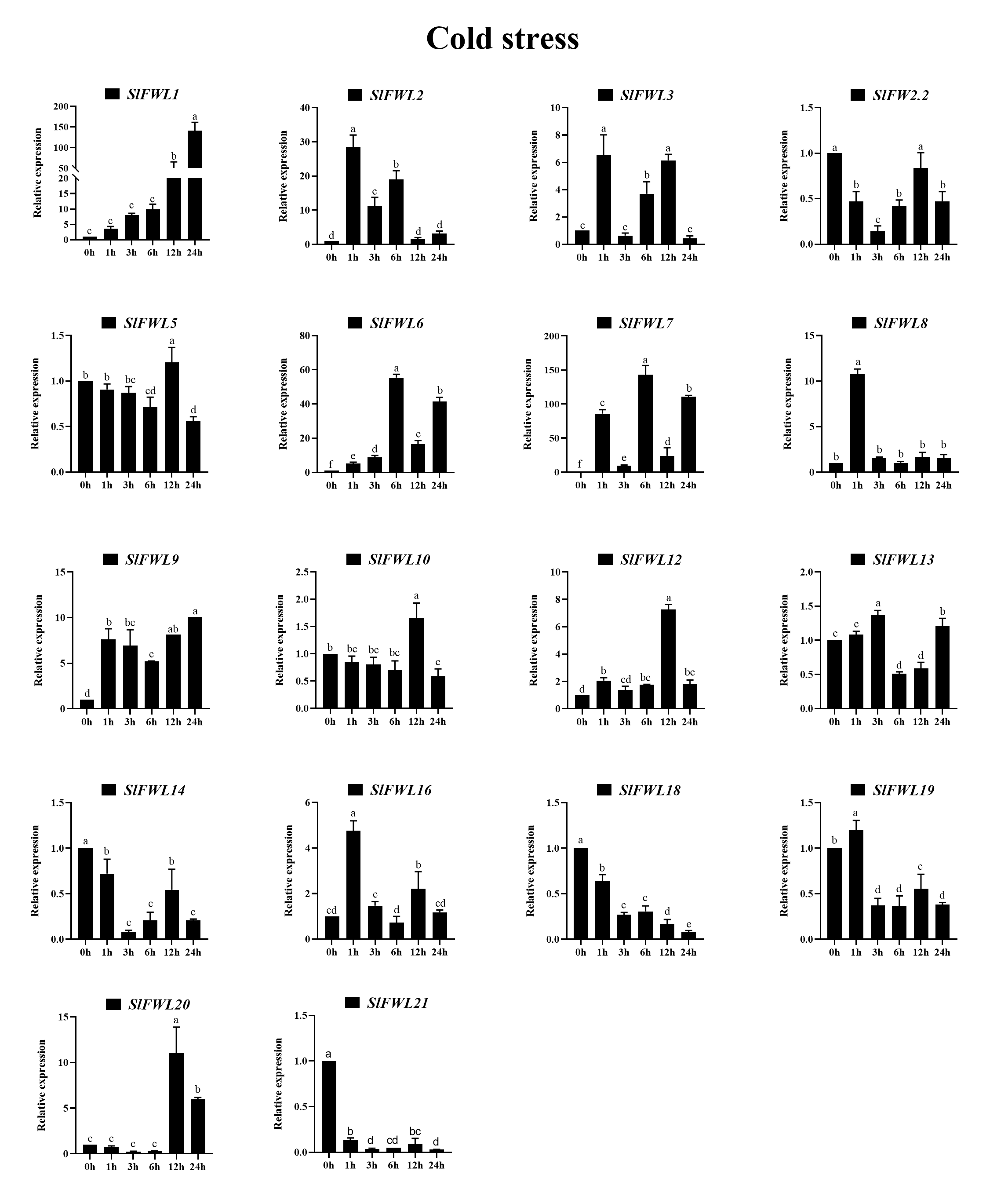

Supplement: Supplementary file 1 [file ijms-24-11783-s001.zip › supplementary/Figure S1.tif]

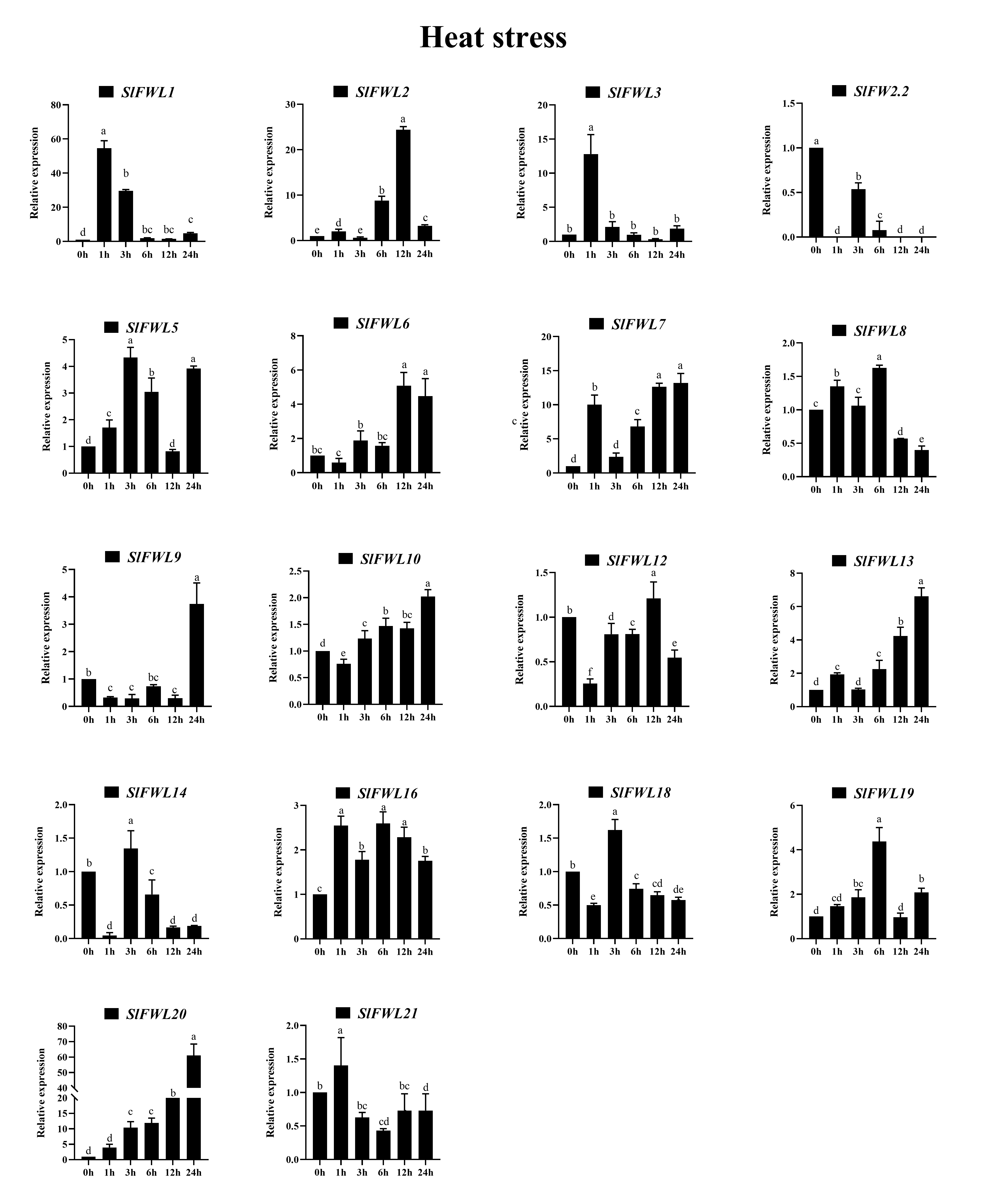

Supplement: Supplementary file 1 [file ijms-24-11783-s001.zip › supplementary/Figure S2.tif]

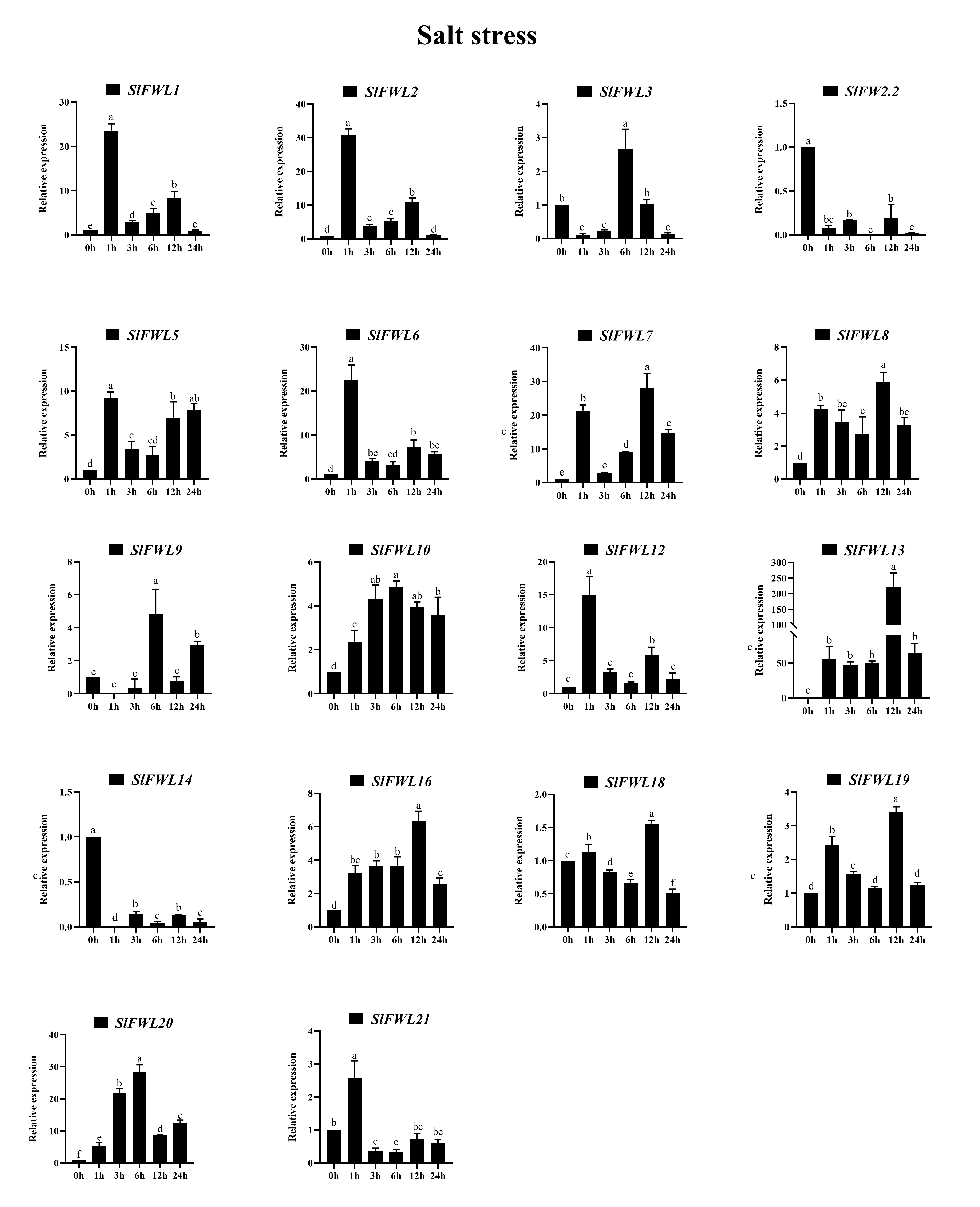

Supplement: Supplementary file 1 [file ijms-24-11783-s001.zip › supplementary/Figure S3.tif]

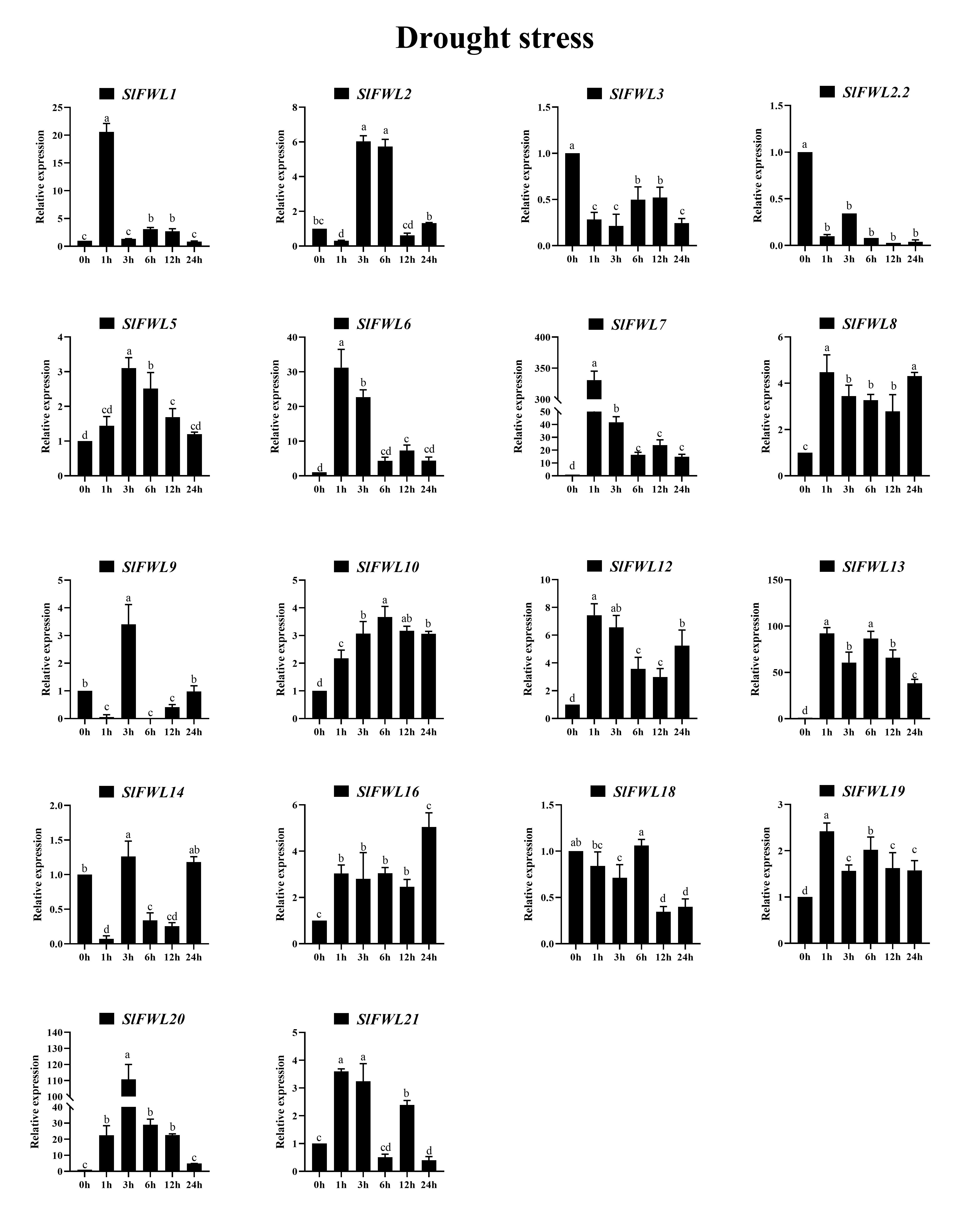

Supplement: Supplementary file 1 [file ijms-24-11783-s001.zip › supplementary/Figure S4.tif]
